# A whole-exome sequencing study of patent foramen ovale: investigating genetic variants and their association with cardiovascular disorders

**DOI:** 10.3389/fgene.2024.1405307

**Published:** 2024-05-14

**Authors:** Xinyi Li, Lingling Xie, Jin Dai, Xinbin Zhou, Tingting Chen, Wei Mao

**Affiliations:** ^1^ The First School of Clinical Medicine, Zhejiang Chinese Medical University, Hangzhou, China; ^2^ Cardiovascular Department, The First Affiliated Hospital of Zhejiang Chinese Medical University (Zhejiang Provincial Hospital of Chinese Medicine), Hangzhou, China; ^3^ Medical Laboratory, The First Affiliated Hospital of Zhejiang Chinese Medical University (Zhejiang Provincial Hospital of Chinese Medicine), Hangzhou, China; ^4^ Department of Cardiology, Affiliated Zhejiang Hospital, Zhejiang University School of Medicine, Hangzhou, China; ^5^ Zhejiang Key Laboratory of Integrative Chinese and Western Medicine for Diagnosis and Treatment of Circulatory Diseases, Hangzhou, China

**Keywords:** patent foramen ovale, whole exome sequencing, pathogenic mutations, functional enrichment analysis, nkx2-5

## Abstract

**Background:**

Patent foramen ovale (PFO) has a genetic predisposition and is closely associated with cryptogenic stroke (CS), migraine, decompression sickness, and hypoxemia. Identifying PFO-related mutant genes through whole-exome sequencing (WES) can help in the early recognition of cardiovascular genetic risk factors, guide timely clinical intervention, and reduce the occurrence of cardiovascular events.

**Methods:**

We analyzed mutant genes from ClinVar and OMIM databases. WES was performed on 25 PFO patients from Zhejiang Provincial Hospital of Chinese Medicine. Pathogenicity of variants was evaluated using American College of Medical Genetics and Genomics (ACMG) and Association for Molecular Pathology. (AMP) guidelines.

**Results:**

In ClinVar (4 Feb 2023), 113 coding gene mutations were found, including 83 associated with PFO. From OMIM (18 Apr 2023), 184 gene mutations were analyzed, with 110 mutant coding genes. WES identified pathogenic mutations in two of 25 PFO patients (8%). LDLR, SDHC, and NKX2-5 genes were linked to PFO and primarily involved in myocardial tissue function. NKX2-5 may play a crucial role in PFO development, interacting with NOTCH1, GATA4, MYH6, SCN5A signaling pathways regulating cardiomyocyte characteristics.

**Conclusion:**

We identified pathogenic mutations in LDLR, SDHC, and NKX2-5 genes, implying their role in PFO development. Functional enrichment analysis revealed NKX2-5’s interaction with signaling pathways regulating cardiomyocyte function. These findings enhance our understanding of PFO’s genetic basis, suggesting potential therapeutic targets for future research.

## 1 Introduction

Patent foramen ovale (PFO) is a common congenital heart disorder that affects approximately 25% of adults worldwide and demonstrating a familial aggregation (Mojadidi et al., 2019). It is characterized by a small hole in the diaphragm of the left and right atria, allowing blood to bypass the pulmonary circulation and flow directly from the right atrium to the left atrium (Homma et al., 2016). This physiological pathway is crucial during fetal development, but if it remains open after 3 years age, it is referred to as PFO. PFO has been associated with various diseases, including migraine, cryptogenic stroke (CS), and decompression sickness (Gillow and Lee, 2015; Lafère et al., 2017; Mojadidi et al., 2021b). CS, as a severe adverse outcome of patent foramen ovale (PFO), is influenced significantly by genetic factors (Bo et al., 2018; He et al., 2018). Research has shown that genetic variants associated with coagulation function, genetic predisposition to cardiac structural abnormalities, and gene variations related to arterial wall elasticity and stability may contribute to the occurrence of CS(Austin et al., 2002; Harland et al., 2002; Cinelli et al., 2019; Lin et al., 2022). These findings highlight the importance of genetic factors in the development of CS as a consequence of PFO. The diagnosis of PFO relies primarily on clinical manifestations and is further supported by complementary examinations, including transesophageal and transthoracic echocardiography (TEE,TTE), as well as transcranial Doppler (TCD) (Liu et al., 2021). There are mainly two treatment options for PFO patients. One option is to use antiplatelet and anticoagulant drugs (such as vitamin K antagonists, direct thrombin inhibitors, or Xa factor inhibitors) to treat PFO complicated stroke (Mas et al., 2017; Kasner et al., 2021). Another method is to insert a special plugging device at the PFO to prevent blood from flowing from the right side of the heart through the ovale foramen into the left atrium (Akobeng et al., 2018). Both treatment modalities have demonstrated efficacy in preventing recurrent ischemic events among patients with cryptogenic stroke and transient ischemic attack (TIA). It has been suggested that heart development, specifically the atrial septum, may be influenced by a combination of different genetic factors rather than a single gene variant (Paolucci et al., 2021). However, PFO patients exhibit variability in onset age, genotype heterogeneity (where one phenotype can be caused by multiple genes), incomplete penetrance, and unclear inheritance patterns. Therefore, molecular genetic testing may offer valuable insights for early diagnosis and subsequent treatment of PFO.

Previous studies have indicated an association between gene polymorphism and PFO patients with CS (Liu et al., 2022). The occurrence of cardiovascular events in PFO patients is closely related to their genetic cardiovascular risk factors. Whole-Exome Sequencing (WES) is an efficient genomic sequencing technique that focuses on analyzing the exonic regions of the genome responsible for encoding proteins. With WES, we aim to identify new genes and rare variations in known genes associated with PFO-related cardiovascular diseases, unravel the genetic complexity of these conditions, and facilitate risk assessment and personalized treatment based on an individual’s genetic profile. This approach may propel the advancement of precision medicine in the field of cardiovascular diseases. The objective of this study is to employ WES technology to identify pathogenic genes, promoter genes, and signaling pathways linked to PFO patients. This will offer more precise guidance for the prevention, diagnosis, and treatment of PFO.

## 2 Materials and methods

### 2.1 Analysis of mutant genes in ClinVar and OMIM databases

To identify PFO-related mutate genes, we searched the terms “patent foramen ovale” in both the ClinVar (https://www.ncbi.nlm.nih.gov/clinvar/) and OMIM (https://omim.org) databases. In the ClinVar database (as of 4 February 2023), we obtained a range of genetic mutation types, including single-allele mutations, multi-copy mutations, non-coding gene deletions, coding gene deletions, and coding gene mutations. In the OMIM database (as of 18 April 2023), we identified gene deletions and gene mutations. We selected mutations in coding genes from both databases for further analysis.

Next, we compared the mutant genes from both databases to find overlapping genes. These overlapping genes were then imported into the String database (https://www.string-db.org) to construct a protein-protein interaction (PPI) network using Cytoscape software. After obtaining gene clusters, we conducted Gene Ontology (GO) and Kyoto Encyclopedia of Genes and Genomes (KEGG) functional enrichment analysis. GO enrichment analysis involves annotating the functions of genes or proteins into different GO terms to identify functional classes associated with diseases or other conditions of interest. KEGG enrichment analysis utilizes pathway annotation information to classify and enrich genes and proteins, providing insights into the pathways associated with specific diseases or biological processes.

### 2.2 Patients

Twenty-five patients with PFO from the cardiovascular department of Zhejiang Provincial Hospital of Chinese Medicine between May 2022 and January 2023 were enrolled for WES, including 20 females and 5 males (Table 1). Right-to-left shunt (RLS) classification criteria: 1) RLS 0, without shunts; 2) RLS 1, 1-9 microbubbles; 3) RLS 2, 10-30 microbubbles; 4) RLS 3, more than 30 microbubbles. All patients signed informed consent forms.

**TABLE 1:** Clinical characteristics of 25 PFO patients.

Characteristics	Simple	Complex	*p*-value
n	17	8	
PFO type, n (%)			<0.001
simple	17 (100.00%)	0 (0.00%)	
long tunnel (length≥8 mm)	0 (0.00%)	6 (75.00%)	
long Euclidean lobe or Hiarli network	0 (0.00%)	1 (12.50%)	
combined atrial septal prolapse tumor	0 (0.00%)	1 (12.50%)	
Years, mean ± sd	46.294 ± 15.459	46.125 ± 15.56	0.98
Migraine, n (%)			0.017
YES	15 (88.24%)	3 (37.50%)	
NO	2 (11.76%)	5 (62.50%)	
Dizzy, n (%)			0.359
NO	13 (76.47%)	4 (50.00%)	
YES	4 (23.53%)	4 (50.00%)	
Syncope, n (%)			1
NO	16 (94.12%)	7 (87.50%)	
YES	1 (5.88%)	1 (12.50%)	
Stroke, n (%)			0.231
NO	16 (94.12%)	6 (75.00%)	
YES	1 (5.88%)	2 (25.00%)	
Hypertension, n (%)			0.283
NO	15 (88.24%)	5 (62.50%)	
YES	2 (11.76%)	3 (37.50%)	
Hyperlipidemia, n (%)			0.059
NO	15 (88.24%)	4 (50.00%)	
YES	2 (11.76%)	4 (50.00%)	
Hyperglycemia, n (%)			1
NO	16 (94.12%)	8 (100.00%)	
YES	1 (5.88%)	0 (0%)	
TTE (Valsaval), n (%)			0.057
RLS I	5 (29.41%)	2 (25.00%)	
RLS II	7 (41.18%)	0 (0.00%)	
RLS III	5 (29.41%)	6 (75.00%)	

Approximately 5 mL of venous blood was collected into Ethylene diamine tetraacetic acid (EDTA) tube from each patient and stored in a −80°C refrigerator for future detection. The diagnostic criteria and evaluation of PFO patients were based on the Chinese Expert Consensus on Ultrasonic Diagnosis of Patent Foramen Ovale. Patients had to meet at least one of the following conditions: 1) Microbubble signals were observed in the left cardiac system within 3-5 cardiac cycles after the right cardiac system was filled with microbubble signals following active saline injection in contrast TTE; 2) Gaps and shunt between the primary and secondary septum were observed in TEE examination; 3) Microbubble signals in the middle cerebral artery were observed within 10 s after intravenous infusion of activated saline in contrast TCD ultrasonography. If the microbubble signal was not detected in the resting state, it was detected when activated saline was administered intravenously again after the Valsalva manoeuvre.

### 2.3 WES

Deoxyribonucleic acid (DNA) was extracted from the venous blood of PFO patients using a DNA blood test kit (Idt xgen exome research panel v1.0). The integrity of the DNA was further assessed by 1% agar gel electrophoresis to determine the degree of DNA degradation and the presence of Ribonucleic acid (RNA) and protein contamination. DNA concentrations were quantified using qubit, and libraries with a minimum of 500 ng could be constructed normally. DNA libraries were prepared by randomly fragmenting DNA into 180-280bp fragments using a nucleic acid interrupter. After terminal repair and A-tail addition, the DNA libraries were ligated to both ends of the fragments. The libraries were then pooled and indexed, followed by hybridization with a biotin-labeled probe in liquid phase. Magnetic beads with streptomycin were used to capture the exons on the gene, and the library was linearly amplified by Polymerase chain reaction (PCR). Once qualified, the library was further quantified using the Qubit instrument and the insert size of the library was determined using Agilent 2100. The effective concentration of the library (3 nM) was accurately quantified using the qPCR method. To ensure the quality of the library, PE150 sequencing was performed on the NovaSeq 6000 platform (Illumina Inc., United States) based on the effective concentration of the qualified library and data production requirements.

### 2.4 Bioinformatics analysis

#### 2.4.1 Screening of pathogenic genes

Offline data analysis process V1.0 for high-throughput sequencing, Human Genome Reference UCSC hg19 February 2009. The application software used for mutation detection is proprietary software self-programmed by Dean Laboratories and BWA. Annotation of variation was made using the public database 1000 Genome (phase I), gnomAD database, variation database ClinVar, dbNSFP, and the proprietary databases LOVD and ZJU-DB. The naming convention for variations is based on Human Genome Variation Society (HGVS).

#### 2.4.2 Variation analysis

The Rare Exome Variant Ensemble Learner (REVEL) (Ioannidis et al., 2016), ClinPred (Alirezaie et al., 2018), Sorting Intolerant FromTolerant (SIFT) (Kumar et al., 2009) and Polymorphism Phenotyping v2 (PolyPhen2) (Adzhubei et al., 2013) were used in combination to analyze the harmfulness of mutation sites. REVEL and ClinPred analyze the pathogenicity of mutation sites, while SIFT and PolyPhen-2 predict the pathogenicity of the encoded proteins. Based on the predictions from these software tools, and in conjunction with the patient’s clinical information, the pathogenicity of the patient is classified according to the American Society for Medical Genetics and Genomics (ACMG) and the American Society for Molecular Pathology (AMP) guidelines (Richards et al., 2015).

REVEL is an integrated method for predicting missense mutations. It combines the prediction results of multiple software tools, including MutPred, FATHMM, VEST, PolyPhen, SIFT, PROVEAN, Mutation Assessor, Mutation Taster, LRT, GERP, SiPhy, phyloP, and phastCons. The method utilizes pathogenic and rare neutral nonsense mutations for training and employs the random forest algorithm, resulting in improved prediction efficacy for rare missense mutations. The predicted score for a single mutation range from 0 to 1, with a score >0.75 generally indicating harm. ClinPred is a prediction tool to identify Disease-Relevant Nonsynonymous Single-Nucleotide Variants. It predicts the harm of mutation by integrating two machine learning algorithms: random forest (cforest) and gradient boosting decision tree (xgboost). The predicted score range for ClinPred is from 0 to 1, with scores above 0.5 predicted as deleterious and scores below 0.5 predicted as harmless. The higher the score, the stronger the pathogenicity of the variation. SIFT predicts the impact of a missense replacement on protein function, thereby determining the harmfulness of the amino acid substitution. SIFT scores range from 0 to 1, with scores less than 0.05 indicating a deleterious effect, while scores greater than or equal to 0.05 suggest no significant harm. PolyPhen2, based on the HumDiv database, is commonly used for complex diseases. A score of 0.957 or higher is predicted to be harmful, a score between 0.453 and 0.956 is predicted to be potentially harmful, and a score of 0.452 or lower is predicted to be harmless.

#### 2.4.3 Pathogenicity rating

The pathogenicity rating of the variation was determined by conducting a literature search and querying databases. Pathogenicity rating refers to the classification standards and guidelines set by the ACMG and AMP, which include categories such as pathogenic, likely pathogenic, benign, likely benign, and of uncertain significance (Richards et al., 2015).

## 3 Results

### 3.1 PFO-related mutant genes in ClinVar and OMIM databases

**FIGURE 1:**
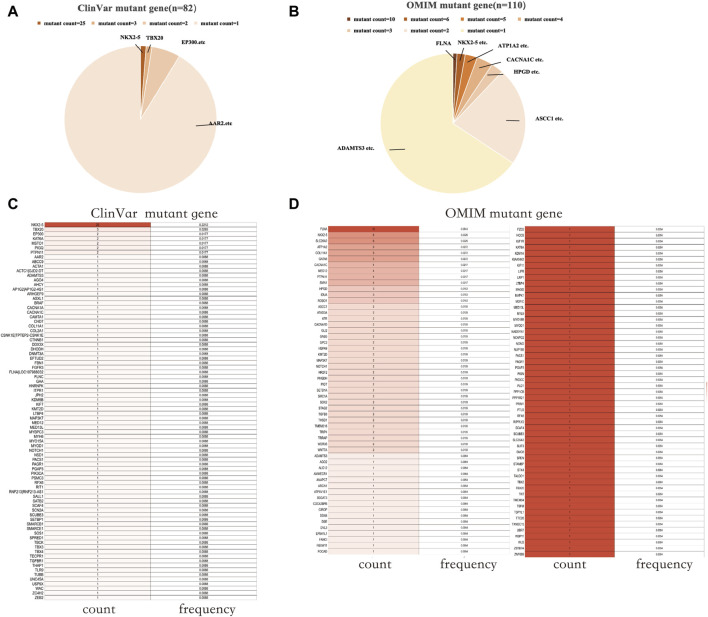
PFO-related mutant genes in public databases. **(A)** Mutant count of PFO-related mutant genes in the ClinVar database. **(B)** Mutant count of PFO-related mutant genes in the OMIM database. **(C)** The count and frequency of mutations in PFO-related mutant genes in the ClinVar database. **(D)** The count and frequency of mutations in PFO-related mutant genes in the OMIM database. PFO: Patent foramen ovale.

The ClinVar database (as of 4th February 2023) yielded 268 results when searching for “patent foramen ovale”. An analysis was conducted on the mutated coding genes, resulting in the detection of 82 distinct gene mutations. The total number of gene mutations observed was 113, with NKX2-5 being the gene showing the highest mutation frequency (25/113) (Figures 1A, C). In the OMIM database (as of 18th April 2023), a search for “patent foramen ovale” generated 116 results. Among these, 190 results indicated gene mutations, including 6 cases of gene deletions and 184 cases of gene mutations. Analysis of the mutant genes revealed the presence of 110 different gene mutations, with FLNA (10/110) and NKX2-5 (6/110) being the genes displaying relatively higher mutation frequencies (Figures 1B, D).

### 3.2 GO and KEGG functional enrichment analysis

**FIGURE 2:**
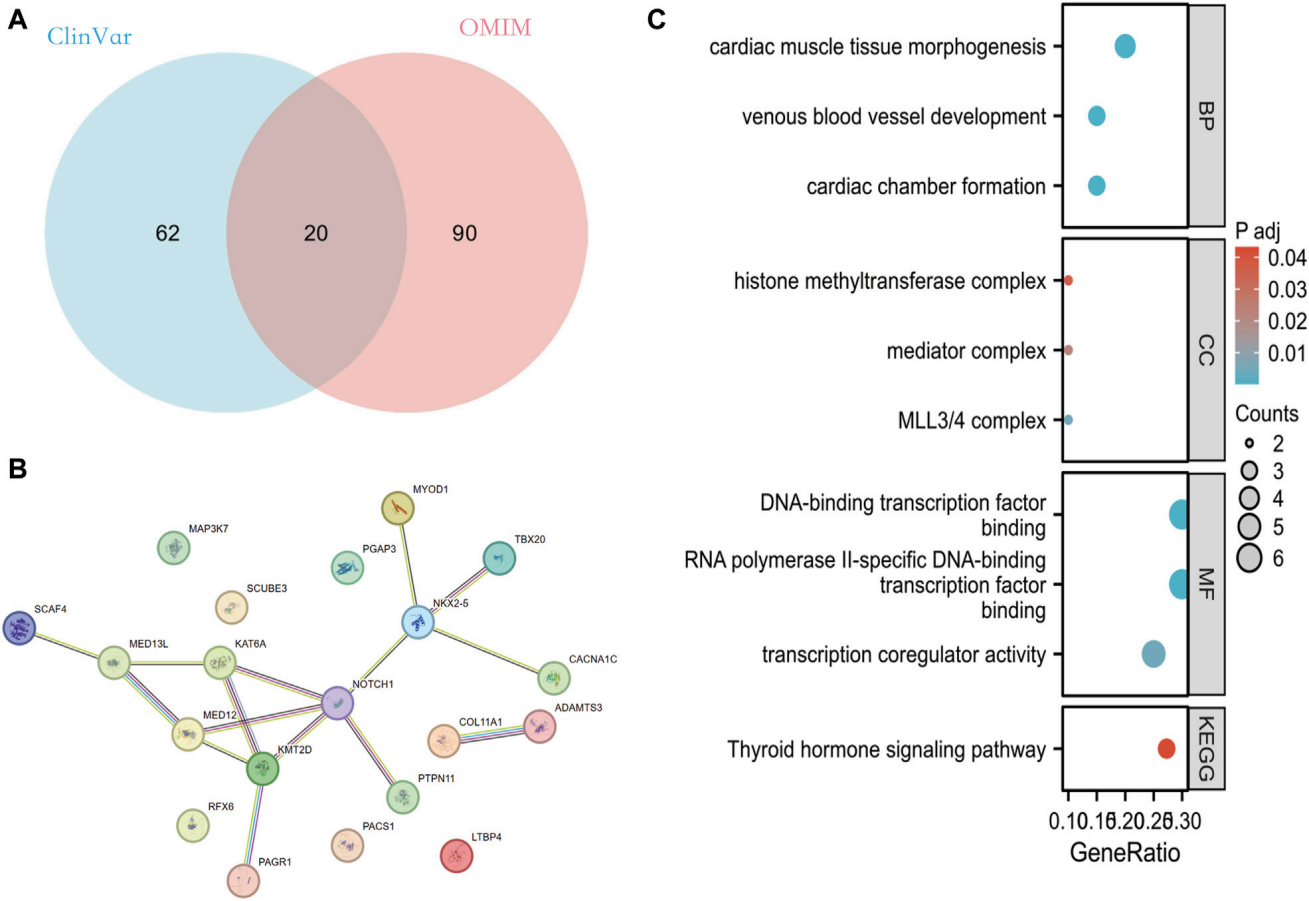
Analysis of mutant genes associated with PFO in ClinVar and OMIM database. **(A)** Overlapping mutant genes related to PFO in the ClinVar and OMIM databases. **(B)** PPI network of these 20 overlapping genes built through String database. **(C)** The GO and KEGG pathway enrichment analysis of these 20 genes. PFO: Patent foramen ovale; PPI: protein-protein interaction network; GO: Gene Ontology; KEGG: Kyoto Encyclopedia of Genes and Genomes.

Venn performed an intersection of the mutant genes from both databases, resulting in the identification of 20 overlapping genes associated with PFO (Figure 2A). By analyzing the constructed protein-protein interaction (PPI) network, we discovered several genes potentially implicated in PFO formation. These genes include NKX2-5, TBX20, KAT6A, PTPN11, CACNA1C, KMT2D, PAGR1, SCAF4, MED12, MED13L, MYOD1, NOTCH1, COL11A1, and ADAMTS3 (Figure 2B).

Enrichment analysis of mutant genes in these two-database revealed that the effects of these genes primarily involve ventricular formation, myocardial tissue morphogenesis, and venous vessel development at the biological level. At the cellular component level, these genes are associated with MLL3/4 complex, histone mediator complex, methyltransferase complex, and more. Furthermore, at the molecular functional level, these genes are involved in RNA polymerase II specific DNA-binding transcription factor binding, DNA-binding transcription factor binding, transcriptional coregulatory activity, and others (Figure 2C).

### 3.3 Identification of gene pathogenicity through WES

Through WES, a total of 48 different mutant genes were detected in the blood samples of 25 patients with PFO, involving 59 mutation sites. The analysis of these gene variations yielded three categories: primary findings, secondary findings, and other potential variations. The primary findings were that the mutant genes were highly relevant to the patient’s clinical information and can be reasonably explained, with sufficient evidence of pathogenicity. The secondary findings were that the mutant genes were highly relevant to the patient’s clinical information and can be reasonably explained, although the evidence for pathogenicity was not robust, but the possibility of pathogenicity was not ruled out. Other potential variations were that the mutant genes were related or possibly related to the patient’s clinical information but cannot be reasonably explained.

In the primary findings, two genes with two specific mutation sites were identified as potentially associated with patent foramen ovale. These genes are LDLR (c.947A>G) and SDHC (c.412dup) (Table 2). The LDLR gene is involved in cellular cholesterol metabolism and has implications in familial hypercholesterolemia, while the SDHC gene is linked to the tricarboxylic acid cycle within the mitochondria. The secondary findings revealed that 42 mutant genes (87.5%) were classified as variants of uncertain significance in terms of clinical relevance (Table 3). In addition to these variations, five other potential mutations (10.4%) were also identified (Table 4). Among the 59 mutation sites we detected, we identified 12 novel mutation sites that are not recorded in the ClinVar database and have not been observed in the East Asian population. We utilized the REVEL and ClinPred software to score and predict the potential pathogenicity of these mutation sites. Following this, we employed SIFT and Polyphen2 to forecast their impact on protein function (Table 5).

**TABLE 2:** The primary findings related to PFO.

Gene	Number of people	Nucleotide change (amino acid change)	Zygotism	ACMG classification
LDLR	1	c.947A>G (p.Asn316Ser)	heterozygous	Likely pathogenic
SDHC	1	c.412dup (p.Asp138GlyfsTer69)	heterozygous	Likely pathogenic

The primary findings: mutant genes were highly relevant to the patient’s clinical information and can be reasonably explained, with sufficient evidence of pathogenicity.

**TABLE 3:** The secondary findings related to PFO.

Gene (n = 42)	Number of people	Transcript number	Nucleotide alteration
ABCC6	1	NM_001171.5	c.2633C>T
ABCG2	1	NM_004827.3	c.376C>T
ACE	1	NM_000789.4	c.945 + 6C>T
AKAP9	1	NM_005751.5	c.3532 + 7T>C
AKT2	1	NM_001626.6	c.441 + 16A>G
APOB	1	NM_000384.3	c.3404G>A
CACNA1A	1	NM_001127221.2	c.4391 + 19A>G
CELA2A	1	NM_033440.3	c.760C>T
CITED2	1	NM_006079.5	c.769A>G
CLCN2	1	NM_004366.6	c.694-11A>C
COL4A1	1	NM_001845.6	c.3506-13C>G
DMD	1	NM_004006.3	c.1704 + 16G>T
DSC2	1	NM_024422.6	c.2488A>C
ELN	1	NM_001278929.2	c.2101T>C
EPAS1	1	NM_001430.5	c.1444-5C>G
EPOR	1	NM_000121.4	c.52C>G
ESR1	1	NM_001122740.2	c.433G>A
EYA4	1	NM_00410.5	c.978C>G
FLNC	2	NM_001458.5	c.5791C>T
		NM_001458.5	c.6527G>A
FLT4	1	NM_182925.5	c.2406 + 16C>A
GATA4	1	NM_002052.5	c.49G>A
HCCS	1	NM_005333.5	c.560G>C
LAMA4	1	NM_002290.5	c.292T>C
LRP6	1	NM_002336.3	c.268G>A
MYBP3	1	NM_000256.3	c.1458-7C>T
MYH11	1	NM_002474.3	c.3616G>A
MYH6	1	NM_002471.4	c.5491G>A
NKX2-5	1	NM_004387.4	c.257T>C
NOTCH1	2	NM_017617.5	c.164C>T
		NM_17617.5	c.562C>G
NOTCH3	3	NM_000435.3	c.6604G>A
		NM_00435.3	c.3371A>G
		NM_00435.3	c.4039G>C
POBO4	1	NM_001301088.2	c.244 + 12G>A
PPARC	1	NM_015869.5	c.328C>T
PRDM16	2	NM_022114.4	c.37 + 15C>T
		NM_022114.4	c.686C>T
PSEN2	1	NM_000447.3	c.893T>C
RNF213	3	NM_001256071.3	c.10424-3C>T
		NM_001256071.3	c.10424-3C>T
		NM_001256071.3	c.12805C>T
		NM_001256071.3	c.12942 + 15C>T
		NM_001256071.3	c.13186-18_13186-14dup
		NM_001256071.3	c.1978C>T
RYR2	1	NM_00103	c.4990G>A
SCN5A	1	NM_198056.3	c.1975C>T
SCNN1B	1	NM_000336.6	c.1074C>A
SDHD	1	NM_003002.4	c.205G>A
TNNI3K	1	NM_015978.3	c.1153T>C
VCL	1	NM_014000.3	c.2191A>G
WBP11	1	NM_016312.3	c.1889A>G

The secondary findings: mutant genes were highly relevant to the patient’s clinical information and can be reasonably explained, although the evidence for pathogenicity was not robust, but the possibility of pathogenicity was not ruled out.

**TABLE 4:** Other potential variations related to PFO.

Gene	Number of people	Transcript number	Nucleotide change
G6PC1	1	NM_000151.4	c.361A>G
SERPINA1	1	NM_000295.5	c.187C>T
NOTCH1	1	NM_017617.5	c.1214C>T
FOXC1	1	NM_001453.3	c.1304C>G
EXPHX2	1	NM_001979.6	c.910 + 4G>A

Other potential variations: mutant genes may be related or potentially related to the clinical information of the clinical subjects, but they cannot be reasonably explained in the context of the subjects’ clinical information.

**TABLE 5:** Novel mutation sites not included in the ClinVar database and not detected in East Asian populations.

Gene	Transcript number	Nucleotide alteration	Amino acid change	REVEL	ClinPred	SIFT	PolyPhed-2
MYH11	NM_002474.3	c.3616G>A	p.Glu1206Lys	0.653	0.985	Harmless	Potentially harmful
NOTCH1	NM_17617.5	c.562C>G	p.Leu188Val	0.176	0.051	Harmless	Harmless
ABCC6	NM_001171.5	c.2633C>T	p.Ser878Phe	0.218	0.105	Harmless	Harmless
WBP11	NM_016312.3	c.1889A>G	p.Tyr630Cys	0.492	0.995	Harmful	Potentially harmful
HCCS	NM_005333.5	c.560G>C	p.Gly187Ala	0.957	/	Harmful	Harmful
ELN	NM_001278929.2	c.2101T>C	p.Phe701Leu	0.034	0.782	Harmful	Harmful
EPOR	NM_000121.4	c.52C>G	p.Leu18Val	0.342	0.782	Harmful	Harmful
NOTCH3	NM_000435.3	c.6604G>A	p.Val2202ILE	0.135	0.005	Harmless	Harmless
GATA4	NM_002052.5	c.49G>A	p.Ala17Thr	0.358	0.534	Harmless	Harmless
LAMA4	NM_002290.5	c.292T>C	p.Cys98Arg	0.948	1.000	Harmful	Harmful
NOTCH3	NM_00435.3	c.3371A>G	p.Asp1124Gly	0.688	0.938	Harmful	Harmful
NOTCH1	NM_017617.5	c.164C>T	p.Pro55Leu	0.030	0.060	Harmful	Harmless

REVEL, rating: >0.750, predicted to be harmful; ClinPred rating: >0.500, predicted to be harmful.

### 3.4 Functional enrichment analysis of mutant genes in PFO patients

**FIGURE 3:**
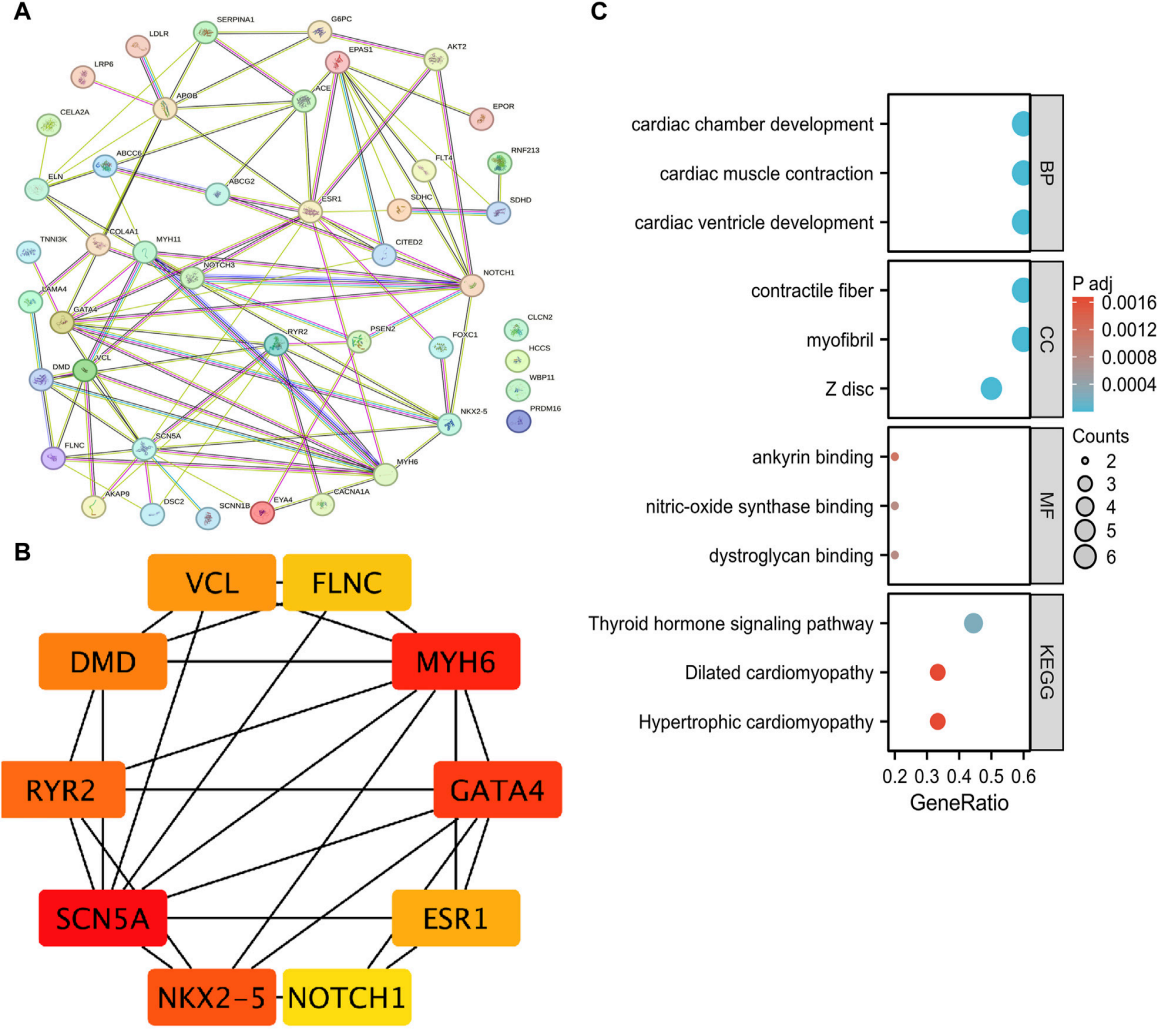
Mutation genes analysis of whole exome detection in 25 clinical PFO patients. **(A)** PPI network of mutation genes built through String database. **(B)** The top ten genes in PPI network obtained using Cytoscape software and MCC algorithm. **(C)** The GO and KEGG pathway enrichment analysis of these 10 genes. PFO: Patent foramen ovale; PPI: protein-protein interaction network; GO: Gene Ontology; KEGG: Kyoto Encyclopedia of Genes and Genomes.

We constructed a protein-protein interaction (PPI) network (Figure 3A) using a String database for these 48 different genes. To further understand the interactions among these genes, we applied the MCC algorithm in Cytoscape software to select the top 10 genes. It is noteworthy that the genes NKX2-5, SCN5A, MYH6, and GATA4 are the most important genes associated with PFO disease (Figure 3B). Functional enrichment analysis was performed on these 10 genes, revealing their significant involvement in cardiac cell biology functions. In terms of molecular function, these genes were found to potentially influence various protein bindings, including calcium-binding proteins, glycoproteins, myosin, and dystrophin. Furthermore, the analysis suggested their potential involvement in several KEGG pathways, such as thyroid hormone signaling, hypertrophic cardiomyopathy, dilated cardiomyopathy, adrenergic signaling in cardiac cells, as well as their association with viral myocarditis (Figure 3C).

## 4 Discussion

PFO, the most common congenital heart abnormality in adults, has been found to have a higher incidence in stroke patients compared to healthy individuals (Gonzalez and Testai, 2021). Nearly half of CS patients have PFO. Research by Del Sette M et al. suggests that PFO occurrence is characterized by family aggregation, indicating the presence of genetic abnormalities (Del Sette et al., 2004). The prevalence of PFO in siblings of young patients with ischemic stroke (IS) is three times greater than in siblings of patients without PFO (Limborska and Filippenkov, 2022). Hereditary hypercoagulability has also been proven to be an important risk factor for PFO-related CS (Harland et al., 2002). In our study, we performed WES to identify mutant genes in 25 PFO patients with clinical symptoms who visited our hospital. Patient outcomes among those with PFO exhibit significant variability. The risk of cardiovascular events in PFO patients is closely related to the anatomical characteristics of the PFO, their genetic background, and the presence of other cardiovascular risk factors (Mojadidi et al., 2021a). In our cohort of 25 PFO patients, 3 cases experienced a stroke, all at an age younger than 45 years. In one of these patients, a suspected pathogenic gene variant was detected (LDLR NM_00527.5 c.947A>G). This mutation results in the substitution of asparagine (Asn) with serine (Ser) at the 316th amino acid of the LDLR protein, which is associated with Familial Hypercholesterolemia (FH). FH leads to abnormally elevated levels of low-density lipoprotein cholesterol (LDL-C) in the blood, increasing the risk of cardiovascular diseases. Mary F Lopez found that in PFO-stroke patients, there was a slight increase in HDL (high-density lipoprotein) and a decrease in cholesterol levels following the closure of the PFO (Lopez et al., 2015). Detecting genetic mutations associated with PFO can help to gain a deeper understanding of the pathogenesis of PFO, the interaction between genes and environmental factors, determine whether patients have genetic risk, and develop personalized treatment plans for patients.

In the ClinVar and OMIM databases, 82 and 110 PFO-related genes were identified, with 20 genes belonging to overlapping genes. Through functional enrichment of 20 overlapping genes, these genes mainly focusing on ventricular formation, myocardial tissue morphogenesis, and venous vessel development at the biological process (BP) level. At the cellular component (CC) level, these genes are related to MLL3/4 complex, histone mediator complex, methyltransferase complex, and others. At the molecular functional (MF) level, these genes are associated with RNA polymerase II specific DNA-binding transcription factor binding, DNA-binding transcription factor binding, transcriptional coregulatory activity, and more. Most of these mutant genes are closely related to the development of heart structure, and they may lead to PFO formation by affecting heart development.

We performed WES on 25 patients with clinical symptoms undergoing PFO closure and identified mutations in 48 related genes. Among these genes, LDLR and SDHC were considered as suspected pathogenic genes. The LDLR gene is primarily involved in cholesterol metabolism at the cellular level, while the SDHC gene is mainly involved in mitochondrial tricarboxylic acid circulation. Furthermore, the SDHC gene has also been linked to gastrointestinal stromal tumor and paraganglioma, although these variants are potential pathogenic factors. Additionally, other pathogenic variants, G6PC1 and SERPINA1 genes, were found. G6PC1 is a pathogenic variant associated with glycogen storage disease type 1a, and SERPINA1 is a suspected pathogenic variant associated with α1-antitrypsin deficiency. Moreover, enrichment analysis revealed that the genes in PFO patients were mainly related to the biological function of muscle tissue, particularly the myocardium. At the cellular level, these genes were associated with myofibril and contractile fiber cells. At the molecular function level, the genes were found to be involved in triosan binding, nitric oxide synthase binding, ankyloprotein binding, actin binding, calmodulin binding, and more. In terms of KEGG pathways, these genes were associated with thyroid hormone signaling pathways, hypertrophic cardiomyopathy, dilated cardiomyopathy, and viral myocarditis.

The analysis of mutation genes in databases and clinical data revealed that NKX2.5 has the highest mutation frequency and has been detected in ClinVar, OMIM, and clinical data. According to the PPI network, NKX2.5 has protein interactions with CACNA1C, MYOD1, TBX20, GATA4, MYH6, RYR2, SCN5A, and NOTCH-1. Cao Y‘s study in 2016 suggested that a variation in the single nucleotide site of NKX2-5 may be linked to the occurrence of Atrial Septal Defect (ASD) (Cao et al., 2016). NKX2-5 regulates the proliferation, migration, differentiation, and function of cardiomyocytes through signaling pathways involving GATA4, MYH6, and others (Välimäki et al., 2017; Wang et al., 2020; Andreasen et al., 2022). Furthermore, several studies have suggested that NOTCH-1 mutation genes are among the most prevalent causes of Congenital Heart Disease (CHD) (Samira et al., 2020; Sarah et al., 2022). Josef Finsterer et al. state that transcription factors NKX2-5 and NOTCH-1 signaling are involved in the pathogenesis of left ventricular hypertrabeculation (LVHT) (Finsterer and Stöllberger, 2020). These studies further confirm the oligogenic effect in the pathogenic process of PFO.

Identification of PFO-related genetic variations through WES allows for the early detection of high-risk PFO patients, enabling timely interventions to reduce potential complications, such as CS. Furthermore, understanding the specific genetic variations in patients who have already been diagnosed with PFO can aid in the formulation of personalized treatment plans. For instance, patients carrying variations in genes such as NKX2-5 or NOTCH-1 may require more intensive monitoring and targeted therapeutic strategies. Additionally, this information on genetic variations can guide patients in genetic counseling, helping them understand the hereditary risks and family history of the disease. Future research can further explore the impact of these genetic variations on treatment outcomes, thereby providing more precise personalized therapies for PFO patients.

Starting from the analysis of genes can provide a deeper understanding of the formation and pathogenesis of PFO, facilitating the identification of specific pathways for targeted interventions that may lead to effective prevention and non-invasive treatment strategies in the future. However, it is important to acknowledge the limitations of this study, such as the small sample size, which may introduce sampling errors and variations in the distribution of disease-causing genes among the sample subjects. Therefore, future research should consider increasing the sample size and employing direct sequence analysis or whole genome sequencing in combination with clinical diagnosis to achieve the highest detection rate of pathogenic genes.

## 5 Conclusion

In this study, we employed WES to identify potential mutant genes and gene mutation sites in patients with PFO. Subsequently, we analyzed PFO-related mutant genes using ClinVar and OMIM databases and found that NKX2-5 genes are involved in the pathogenesis of PFO through other mutation sites and signaling pathways. The findings of our study provide important insights for genetic counseling and gene therapy in PFO patients.

## Data Availability

The original contributions presented in the study are included in the article/supplementary material, further inquiries can be directed to the corresponding authors.
